# Wound management, healing, and early prosthetic rehabilitation: Part 3 - A scoping review of chemical biomarkers

**DOI:** 10.33137/cpoj.v8i1.43717

**Published:** 2025-02-21

**Authors:** H Williams-Reid, A Johannesson, A Buis

**Affiliations:** 1 Department of Biomedical Engineering, Faculty of Engineering, University of Strathclyde, Glasgow, Scotland.; 2 Össur Clinics EMEA, Stockholm, Sweden.

**Keywords:** Amputation, Scoping Review, Wound Healing, Surgical Site Healing, Chemical Biomarkers, Chemical Markers of Healing, Residuum Healing, Residual Limb Healing, Wound Management, Early Prosthetic Rehabilitation

## Abstract

**BACKGROUND:**

Poor post-amputation healing delays prosthetic fitting, adversely affecting mortality, quality of life, and cardiovascular health. Current residual limb assessments are subjective and lack standardized guidelines, emphasizing the need for objective biomarkers to improve healing and prosthesis readiness assessments.

**OBJECTIVE(S):**

This review aimed to identify predictive, diagnostic, and indicative chemical biomarkers of healing of the tissues and structures found in the residual limbs of adults with amputation.

**METHODOLOGY:**

This scoping review followed Joanna Briggs Institute (JBI) and PRISMA-ScR guidelines. Searches using the terms “biomarkers,” “wound healing,” and “amputation” were performed across Web of Science, Ovid Medline, Ovid Embase, Scopus, Cochrane, PubMed, and CINAHL databases. Inclusion criteria were: 1) References to chemical biomarkers and healing; 2) Residuum tissue healing; 3) Repeatable methodology with ethical approval. Included articles were evaluated for quality of evidence (QualSyst tool) and level of evidence (JBI classification). Sources were categorized by study (e.g., randomized controlled trial or bench research), wound (diabetic, amputation, other), and model (human, murine, other) type. Chemical biomarkers repeated across study categories, and quantification methods were reported on.

**FINDINGS:**

From 3,306 titles and abstracts screened, 646 underwent full-text review, and 203 met the criteria for data extraction, with 76% classified as strong quality. 38 chemical biomarkers were identified across 4 to 50 sources, with interleukins (predictive, indicative, and diagnostic) and HbA1c (predictive) most prevalent, appearing in 50 and 48 sources, respectively. Other biomarkers included predictive blood markers (e.g., cholesterol, white blood cell counts), indicative growth factors, bacteria presence (predictive), proteins (predictive, indicative, and diagnostic, e.g., matrix metalloproteinases), and cellular markers (indicative and diagnostic, e.g., Ki-67, alpha-smooth muscle actin [α-SMA]).

**CONCLUSION:**

Predictive biomarkers identify comorbidities that may hinder healing, aiding in pre-amputation risk assessment for poor recovery. Indicative biomarkers monitor key biological healing processes, such as angiogenesis (the formation of new blood vessels), wound contraction, and inflammation. Diagnostic biomarkers provide direct insights into tissue composition and cellular-level healing. Integrating these biomarkers into post-amputation assessments enables continuous monitoring of the healing process while accounting for comorbidities, enhancing the objectivity of post-surgical healing management and ensuring more effective, personalized rehabilitation strategies.

## INTRODUCTION

### 1: OVERALL RATIONALE, AIMS, AND OBJECTIVES

A wound is defined as damage to biological tissue,^1^ encompassing various forms, including deep tissue injuries associated with prolonged prosthesis use and the surgical site resulting from amputation. The wound healing process is a complex biological process involving four interlinked phases: hemostasis, inflammation, proliferation, and tissue remodeling.^2–4^ This process requires intricate cellular coordination, rendering it vulnerable to impairment that can result in a stalled (also known as chronic or non-healing) wound.^5^ Amputation surgical sites, however, do not always heal optimally, instead experiencing complications such as infection, pain, wound dehiscence, stitch abscesses, tissue necrosis, and poor residual limb formation.^6,7^ These complications stall healing and, in severe cases, necessitate revision surgeries or re-amputation.^6^

Despite their critical role in preventing complications like re-amputation, wound healing assessments remain subjective.^8^ This is particularly significant for individuals with major lower limb amputation, defined as amputation through or proximal to the ankle, whose readiness for prosthetic rehabilitation depends on the health and healing of their residual limb. Early prosthetic fitting improves mobility, ambulation, and daily functioning,^9–11^ whilst increased costs and elevated three-year post-amputation mortality rates are associated with delays or failure to provide timely prosthetic interventions.^10,11^ However, current evaluations of the residual limb post-amputation rely on clinical judgement, lacking standardized guidelines or objective metrics.^8,12,13^ While factors like wound healing, pain management, and limb volume are considered, they are not consistently quantified. Additionally, debates over rehabilitation practices promoting residual limb healing, such as the use of rigid versus soft immediate post-operative dressings,^14,15^ further highlight inconsistencies in clinical approaches.

There is a need for objective measures, such as biomarkers, to evaluate wound healing and thus readiness for prosthetic fitting. Biomarkers, as defined by the United States Food and Drug Administration (U.S. FDA) as measurable indicators of biological processes or responses to treatment,^16^ provide a means to minimize the subjectivity of current practices. However, their application in early-stage post-amputation healing remains largely unexplored.^8,17,18^ To address this research need, a scoping review was developed and implemented with the following aim:

Identify predictive, diagnostic, and/or indicative biomarkers (physical, chemical, or other) of healing of the tissues and structures found in the residual limbs of adults with amputation.

To meet this aim, the following objectives were compiled:

**1**) Collate and synthesize the reported definitions of healing and non-healing in the literature investigating healing of the tissues and structures found in the residual limbs of adults with amputation.
**2**) Identify and collate physical biomarkers predictive, diagnostic, and/or indicative of healing repeated in sources investigating healing of the tissues and structures found in the residual limbs of adults with amputation.
**3**) Identify and collate chemical biomarkers predictive, diagnostic, and/or indicative of healing repeated in sources investigating healing of the tissues and structures found in the residual limbs of adults with amputation.
**4**) Assess the quality and levels of evidence from sources investigating the healing of the tissues and structures found in the residual limbs of adults with amputation.

In the aim, biomarkers are classified by their nature and function. Physical biomarkers are measurable attributes of the wound or tissue itself, such as wound pH or temperature, whilst chemical biomarkers are molecules found in biological tissue or fluids (e.g., sweat, sebum, saliva, and blood) that signal biological processes such as cytokines. Functionally, predictive biomarkers assess the likelihood of a healing state or treatment response, while diagnostic biomarkers definitively confirm healing progression or status. Indicative biomarkers suggest the presence of a condition or physiological state but are not definitive.

### 2: PART 3 RATIONALE, AIMS, AND OBJECTIVES

This article (Part 3) addresses Objective 3 and is the final instalment in a three-part series examining Objectives 1 through 3. Part 1 highlighted significant gaps in defining healing and non-healing, emphasizing the need for an amputation-specific wound healing assessment scale incorporating objective measures like biomarkers.^17^

Part 2 focused on physical biomarkers quantifying macrolevel physiological properties.^18^ While useful and easily noninvasively measured, these biomarkers, such as hemodynamic and oxygenation measures, often indicate changes resulting from cellular healing processes rather than directly representing the healing process itself. For example, wound temperature changes (a physical biomarker) may reflect inflammation, immune responses, vasodilation, and tissue metabolism.^19–21^ In contrast, chemical biomarkers like interleukins and C-reactive protein directly signal inflammatory responses^22^ and serve as more precise diagnostic indicators of healing mechanisms.

Currently, poor healing is defined by clinical endpoints like wound dehiscence or necrotic tissue formation.^17^ Chemical biomarkers provide earlier insights into the healing process, allowing evaluation of treatments and rehabilitation programs. For instance, serum levels of matrix metalloproteinase 2 (MMP-2) and MMP-7 can predict wound dehiscence,^23,24^ as these MMPs support extracellular matrix remodeling, which is essential for tensile skin strength.^25^

Chemical biomarkers provide diagnostic insights into healing because they are intrinsic components of the healing process, with their levels directly reflecting specific healing mechanisms. For instance, the Ki-67 protein functions as a marker of cellular proliferation in human cells.^26,27^ The proliferation of fibroblasts, endothelial cells, and keratinocytes is vital for cutaneous wound healing, as it constitutes the third stage of the four-step healing process.^28^ To demonstrate the indicative and diagnostic power of Ki-67 in healing, Escuin-Ordinas et al.^29^ observed that diabetic wounds with higher wound closure scores exhibited significantly greater numbers of Ki-67-positive cells. Similarly, collagen, another chemical biomarker, is embedded in essential healing mechanisms. It aids healing by attracting fibroblasts and promoting new collagen formation within the wound bed.^30^ Thus, chemical biomarkers offer sensitive, specific measures of healing, improving understanding of post-amputation recovery and guiding rehabilitation. Therefore, the aim of this review was to:

Identify predictive, diagnostic, and/or indicative chemical biomarkers of healing in the tissues and structures found in the residual limbs of adults with amputations.

To achieve this aim, the following objectives were established:

**1)** Identify and compile chemical biomarkers that are predictive, diagnostic, and/or indicative of healing as reported in sources investigating the tissues and structures of residual limbs in adults with amputations.
**2)** Identify and summarize the techniques used to quantify these chemical biomarkers in studies focused on the healing of tissues and structures in residual limbs of adults with amputations.
**3)** Assess the quality and levels of evidence in sources investigating the healing of tissues and structures found in the residual limbs of adults with amputations.

## METHODOLOGY

The detailed methodology and rationale for this review have been outlined previously in Parts 1^17^ and 2.^18^ Briefly, the review adhered to the Preferred Reporting Items for Systematic Reviews and Meta-Analyses extension for Scoping Reviews (PRISMA-ScR) checklist^31,32^ and the Joanna Briggs Institute (JBI) guidelines.^33–36^ Data were managed using Excel Version 2303 (Microsoft, Washington, USA) on Windows 11 Version 22H2 (Microsoft, Washington, USA).

### 1: INCLUSION CRITERIA AND SEARCH STRATEGY

Finalized search terms, based on the terms “biomarker”, “amputation”, and “wound healing”, were applied to Web of Science, MEDLINE (Ovid), Embase (Ovid), Scopus, Cochrane, PubMed, and CINAHL databases. In stage one of screening titles and abstracts were screened using primary inclusion criteria: references to biomarkers of healing and publications from 2017 onward. Given the limited exploration of chemical biomarkers in early-stage post-amputation healing,^8,17,18^ the inclusion criteria were broadened to cover tissues and structures biologically comparable to those in a residuum (e.g., skin, muscles, tendons, ligaments, bone, peripheral nervous system, and vasculature). In the second screening phase of full-texts, additional criteria were introduced, including reproducible methodologies, clear ethical approval (where applicable), and participants aged 18 years or older for human studies. Bench research using in vitro, in silico, or murine models was considered for inclusion to capture biomarkers requiring cell or tissue samples which are ethically easier to obtain in these contexts. Murine models were considered suitable due to sufficient genetic similarities to humans and common use in biological research.^37^ Studies from all contexts and regions were considered if available in English. Search results were managed in EndNote 20 (Version 20.2.1, Clarivate, 2021), where duplicates were removed.

### 2: DATA EXTRACTION, ANALYSIS AND PRESENTATION

Using a pre-defined data extraction tool (Part 1, Appendix A^17^), data (including chemical biomarkers and study characteristics) was extracted from sources that passed both screening rounds. Study quality and evidence levels were evaluated using the QualSyst tool^38^ and JBI levels of evidence^39^ respectively. All extracted data are openly accessible in the review's dataset.^40^

Included sources were categorized by study (randomized controlled trial, case-controlled, observational, or bench research), wound (diabetic, amputation, or other), and model (human, murine, or other) type. Chemical biomarkers that were observed more than once within and across study categories are reported on. These repeated chemical biomarkers are represented in tabular form and analyzed in comparison with existing literature for their indicative, predictive, and/or diagnostic potential in healing assessment. The review emphasizes recurring biomarkers, assuming their repeated observation indicates a stronger evidence base for the biomarker's use, thus supporting future research. Descriptive results section (section 3: Measurement Techniques of The Repeated Chemical Biomarkers) and discussion section (section 2.2: Quantification Techniques) summarize biomarker quantification methodologies, offering additional context for the future use of the biomarkers in residual limb healing management.

## RESULTS

### 1: OVERALL RESULTS

#### 1.1: Search Strategy Results

As disseminated in Part 1,^17^ the search strategy identified 7,041 sources for screening. Of these, 3,735 were duplicates and were subsequently removed (Part 1 - PRISMA diagram). From the remaining 3,306 articles screened at the title and abstract level, 646 met the criteria for full-text screening. 219 articles satisfied the inclusion criteria and were selected for data extraction. Primary reasons for exclusion included unclear methodologies, lack of ethical approval, and review article study type. Of the 219 included sources, 203 reported on chemical biomarkers and are therefore the focus of this Part 3 review.

#### 1.2: Quality and Levels of Evidence

The quality assessment of the included sources revealed a strong emphasis on high-quality quantitative research. The majority of included sources (155 out of 203, or 76%)^29,41–194^ were classified as being of strong quality. An additional 40 sources^195–234^ were rated as good quality, while only 8 sources^235–242^ fell into the adequate quality category. None of the sources were categorized as having limited quality.

Contrastingly levels of evidence of the included sources demonstrated greater variability. In the Prognosis category, 29 studies were classified as level 1.b, representing the second-highest evidence level, while only 3 studies^64,68,173^ fell into level 5.c, the lowest evidence tier (**Table 1**). Conversely, within the Effectiveness category, a minimal number of studies were rated at the higher evidence levels, with 1 study^207^ classified as 1.b and 12 studies^75,109,170,176,182,199,204,205,210,221,222,241^ as 1.c (**Table 1**). However, the majority of studies in this category, (96 studies) were assigned to level 5.c. This prevalence of level 5.c can be attributed to the significant number of bench research studies (Study Categories 9 to 13 in **Table 2**), which are considered the lowest evidence level.

**Table 1:** Levels of evidence of the 203 included articles ranked using the JBI (Joanna Briggs Institute) Levels of Evidence (44) (NA = not applicable).

Evidence Level	JBI Evidence Level Study Categories		
	Effectiveness	Diagnosis	Prognosis
**1.a**	0	0	0
**1.b**	**1** (207)	**7** (67, 73, 85, 130, 154, 217, 239)	**29** (47, 61, 69, 72–74, 85, 86, 90, 115, 116, 120, 123, 130, 138,
**1.c**	**12** (75, 109, 170, 176, 182, 199, 204, 205, 210, 221, 222, 241)	NA	NA
**1.d**	0	NA	NA
**2.a**	0	0	0
**2.b**	0	0	0
**2.c**	0	NA	NA
**2.d**	0	NA	NA
**3.a**	0	0	0
**3.b**	**1** (104)	0	**42** (43, 44, 46, 58, 60, 62, 63, 65, 67, 77–79, 81–84, 92, 94, 100, 102, 106, 128, 129, 131, 133, 134, 140, 142, 147, 150, 160, 168, 171, 172, 179, 181, 185, 200, 220, 228, 230, 235)
**3.c**	**3** (89, 203, 217)	NA	NA
**3.d**	**3** (97, 127, 195)	NA	NA
**3.e**	**30** (43, 46, 48, 50, 52, 57, 59, 62, 67, 73, 74, 85, 90, 94, 99, 129, 131, 143, 144, 146, 149, 164, 175, 185, 189, 198, 224, 236, 238, 240)	NA	NA
**4.a**	0	0	0
**4.b**	0	0	**2** (97, 152)
**4.c**	0	NA	NA
**4.d**	**1** (145)	NA	NA
**5.a**	0	0	0
**5.b**	0	0	0
**5.c**	**96** (29, 41, 42, 45, 49, 51, 53–56, 64, 66, 68, 70, 71, 76, 80, 87, 88, 91, 93, 95, 96, 98, 101, 103, 105, 107, 108, 110–114, 117–119, 121, 122, 124–126, 132, 135–137, 139, 141, 148, 151, 153, 155, 156, 158, 161, 163, 165–167, 174, 177, 178, 180, 183, 184, 186–188, 190–194, 196, 201, 202, 206, 208, 209, 211–216, 218, 219, 223, 226, 227, 229, 231–233, 237, 242)	**3** (68, 126, 194)	**3** (64, 68, 173)

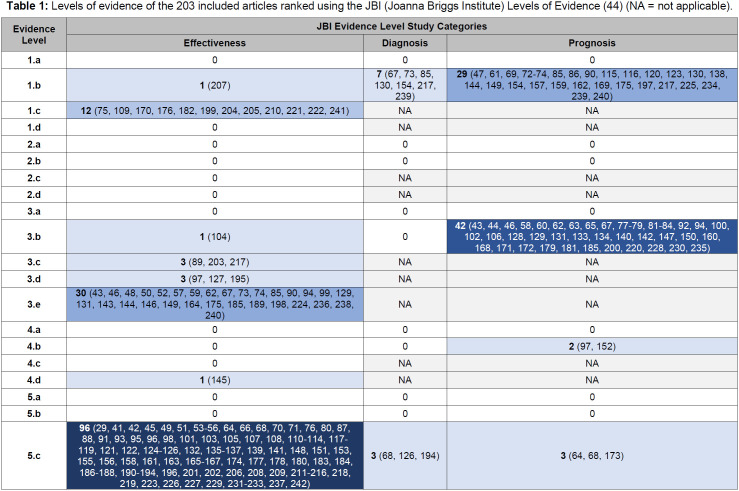

**Table 2:** Summary of the study types of all 203 included sources utilizing chemical biomarkers. The sources are categorized by study type, wound type, and model type, with reference numbers provided for each category as used throughout the review.

Study Type				Category Reference	Number Number (%) of Included Sources	Included Source References
Randomized Controlled Trial				1	13 (6%)	(75, 109, 170, 176, 182, 199, 204, 205, 207, 210, 221, 222, 241)
Case-Controlled Study				2	6 (3%)	(97, 127, 131, 145, 152, 195)
Observational	Prospective	Diabetic Wounds		3	29 (14%)	(47, 48, 59, 61, 62, 69, 72–74, 89, 90, 115, 120, 123, 138, 143, 144, 146, 157, 197, 198, 203, 217, 224, 225, 234, 236, 238, 239)
		Amputation		4	8 (4%)	(57, 85, 130, 149, 159, 162, 169, 240)
		Other Wounds		5	6 (3%)	(50, 52, 86, 99, 154, 175)
	Retrospective	Diabetic Wounds		6	18 (9%)	(58, 63, 67, 81, 84, 92, 94, 100, 133, 134, 164, 171, 172, 179, 181, 185, 230, 235)
		Amputation		7	14 (7%)	(43, 44, 46, 60, 79, 83, 129, 140, 142, 150, 160, 189, 200, 220)
		Other Wounds		8	12 (6%)	(65, 77, 78, 82, 102, 104, 106, 116, 128, 147, 168, 228)
Bench Research		Diabetic Wounds	Rat Models	9	25 (12%)	(51, 54, 70, 88, 96, 110, 111, 113, 117, 121, 122, 125, 136, 137, 165, 167, 174, 186, 191, 218, 223, 229, 231−233)
			Mouse Models	10	41 (20%)	(29, 49, 56, 66, 71, 80, 91, 93, 103, 105, 107, 112, 114, 118, 119, 124, 126, 153, 158, 177, 178, 180, 183, 187, 188, 190, 192–194, 201, 202, 206, 208, 209, 212, 213, 215, 216, 219, 226, 227)
			Other Models	11	14 (7%)	(41, 42, 45, 68, 76, 95, 98, 108, 132, 135, 151, 156, 196, 237)
		Other Wounds	Rat/Mouse Models	12	13 (6%)	(55, 64, 87, 101, 141, 148, 155, 161, 163, 173, 184, 211, 214)
			Other Models	13	4 (2%)	(53, 139, 166, 242)

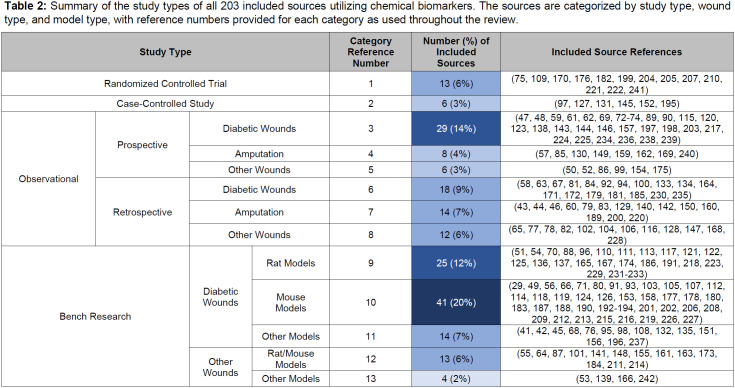

For a comprehensive discussion of the quality and evidence levels of all 219 sources that satisfied the inclusion criteria for the overall review aim, refer to Part 1.^17^

#### 1.3: Study Types and Characteristics

Of the 203 included sources, observational and bench research studies accounted for the largest proportions, compromising 89 and 97 sources, respectively (**Table 2**). In contrast, only 13 sources were randomized controlled trials (RCTs), and 6 were case-controlled studies.

In Categories 1 to 8 (**Table 3**), studies involving human participants featured sample sizes ranging from just 1 participant in a case-controlled study^145^ to 11,943,000 participants in an observational retrospective study.^140^ This large sample size is attributed to the examination of annual rates of hemoglobin A1c (HbA1c) testing and major leg amputations among Medicare patients with diabetes spanning from 2003 to 2012 across 306 hospital referral regions in the USA.^140^ Of the 106 sources, 95 provided gender information, with median male representation within each category ranging from 50% to 71% of participants (**Table 3**).

**Table 3:** The characteristics (wound type, sample size, gender distribution, and age) of the included sources involving human participants (Study Categories 1 to 8; **Table 2**). Note that for wound type some sources fall under more than one wound type. For example, Norvell et al.^142^ (a Category 7 source) investigated wound healing of lower limb amputation due to diabetes or peripheral arterial disease. The notation “No. (%) of references” indicates the number and percentage of sources that provide characteristic information relative to the total number of sources within that category (T.G. = treatment groups; C.G. = control groups; No. = number; NA = not applicable).

	Study Category							
	**1**	**2**	**3**	**4**	**5**	**6**	**7**	**8**
**Wound Type Totals**								
Diabetic	**10** (75, 170, 176, 182, 199, 205, 207, 221, 222, 241)	**5** (127, 131, 145, 152, 195)	**29** ^*^	**1** (159)	0	**18** ^*^	**5** (83, 129, 140, 142, 200)	0
Amputation	**1** (109)	**4** (97, 131, 145, 152)	0	8^*^	0	0	**14** ^*^	0
Other	**2** (204, 210)	0	0	0	6^*^	0	0	**12** ^*^
**Sample Size Totals**								
Range (Min-Max)	15–200	1–120	4–684	10–556	5–735	48–1032	46–11943000	45–637
Median	33	20	57	21	18	148	205	125
No. (%) of References	**13** (100%)	**6** (100%)	**29** (100%)	**8** (100%)	**6** (100%)	**18** (100%)	**14** (100%)	**12** (100%)
**Sample Gender (% Male) Totals**								
Range (Min-Max)	40%–82%	0%–100%	33%–91%	55%–100%	20%–79%	44%–85%	45%–99%	54%–82%
Median	63%	50%	63%	64%	62%	64%	71%	67%
No. (%) of References	**11** (85%) (109, 170, 176, 182, 199, 204, 205, 207, 210, 222, 241)	**6** (100%)	**26** (90%) (47, 48, 59, 61, 62, 72–74, 89, 90, 115, 120, 123, 138, 143, 144, 146, 157, 197, 198, 203, 217, 224, 225, 234, 239)	**8** (100%)	**4** (67%) (52, 86, 99, 175)	**15** (83%) (58, 63, 67, 81, 84, 92, 94, 100, 164, 171, 172, 181, 185, 230, 235)	**14** (100%)	**11** (92%) (65, 77, 78, 82, 102, 104, 106, 116, 128, 147, 168)
**Sample Mean Age (Years) Total**								
Range (Min-Max)	T.G.: 40.6–69.0; C.G.: 28.8–64.7	60.2–65.0	47.4–73.4	49.0–74.0	NA	54.5–72.5	38.0–77.3	56.0–74.0
Median	T.G. 58.1;C.G.: 58.9	61.5	59.5	65.2	NA	61.2	66.7	72.0
No. (%) of References	**12** (92%) (75, 109, 170, 176, 182, 199, 204, 205, 207, 210, 222, 241)	**3** (50%) (97, 127, 145)	**27** (93%) (47, 48, 59, 61, 62, 69, 72–74, 89, 90, 115, 120, 123, 138, 143, 144, 146, 157, 197, 198, 203, 217, 224, 225, 234, 239)	**6** (75%) (85, 149, 159, 162, 169, 240)	NA	**16** (89%) (58, 63, 67, 81, 84, 92, 94, 100, 133, 134, 164, 171, 181, 185, 230, 235)	**12** (86%) (43, 46, 60, 83, 129, 140, 142, 150, 160, 189, 200, 220)	**8** (67%) (65, 77, 78, 102, 104, 106, 147, 168)
**Sample Age Range (Years) Total**								
Range (Min-Max)	NA	35–94	20–89	23–87	28–88	23–100	26–96	22–96
No. (%) of References	NA	**3** (50%) (131, 152, 195)	**11** (38%) (48, 61, 69, 90, 115, 138, 143, 198, 203, 217, 225)	**3** (38%) (57, 85, 159)	**4** (67%) (52, 86, 99, 175)	**6** (33%) (63, 84, 94, 133, 134, 235)	**2** (14%) (43, 129)	**1** (8%) (147)
**Sample Median Age (Years) Totals**								
Range (Min-Max)	NA	NA	NA	NA	NA	NA	47.0–62.0	31.0–71.2
Median	NA	NA	NA	NA	NA	72.5	54.5	68.4
No. (%) of References	NA	NA	NA	NA	NA	**1** (6%) (172)	**2** (14%) (44, 79)	**3** (25%) (82, 116, 128)

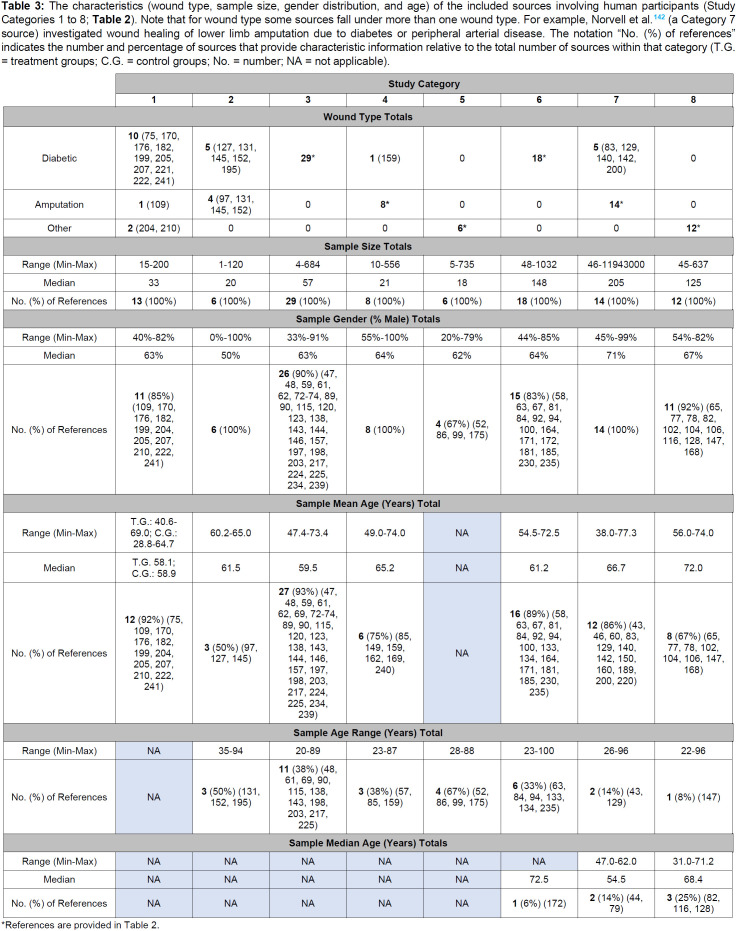

* References are provided in Table 2.

Median mean participant ages exceeded 58 years across all study categories, with reported means ranging from 28.8^204^ to 77.3^220^ years. Among the human participant studies, 68 investigated diabetic wounds, 27 focused on amputations (some resulting from diabetic wounds), and 20 explored other wound types (**Table 3**). Examples of the latter included skin wounds,^50,175^ lower limb mangled extremities,^154^ and infected wounds.^128^

Similarly, to the human participant studies, bench research predominantly used male subjects and focused on diabetic wounds. Of the murine models employed in 81% (79 sources) of the bench research studies, 56 sources utilized all male rats/mice, 6 sources used all female, and the remaining 17 sources used both or did not specify gender (**Table 2**). In place of murine models, the remaining bench research studies utilized cell lines and tissue samples (15 sources^41,42,45,53,95,98,108,132,135,139,151,166,196,237,242^), a mathematical model (1 source^76^), and a gene expression dataset (1 source^156^). Categories 9 to 11 (80 sources; **Table 2**) specifically investigated diabetic wounds, while 7,^55,101,141,148,184,211,214^4,^64,161,163,173^ 2,^87,155^ and 4,^53,139,166,242^ sources explored skin wounds, traumatic injuries, sciatic nerve injuries, and wound/scratch assays (a type of cellbased wound model), respectively.

### 2: REPEATED CHEMICAL BIOMARKERS

Of 38 identified repeated chemical biomarkers (**Table 4**), interleukins (ILs) were the most frequently reported, appearing in 50 sources (25% of 203 included sources). This was followed by glycated hemoglobin (HbA1c) and vascular endothelial growth factor (VEGF), which were utilized in 48 and 39 sources, respectively. Other notable biomarkers included C-reactive protein (CRP) and tumor necrosis factor (TNF), each reported in 34 studies, and albumin, which was employed in 31 sources. Biomarkers such as matrix metalloproteinases (MMPs), collagen, and creatinine were observed in 10% to 14% of sources, whereas less frequently reported biomarkers, including zinc and myeloperoxidase (MPO), were present in only 2% to 5% of studies.

**Table 4:** A comprehensive breakdown of the repeated chemical biomarkers. A biomarker was considered “repeated” if it was used in more than one source within a study category and appeared in more than one study category. The occurrence of these biomarkers in the 203 included sources is presented, along with their representation across the various study categories (**Table 2**; freq. = frequency; ILs = interleukins; HbA1c = glycated hemoglobin; VEGF = vascular endothelial growth factor; CRP = C-reactive protein; TNF = tumor necrosis factor; TGF = transforming growth factor; WBC = white blood cells; CD = cluster of differentiation; Hb = hemoglobin; α-SMA = alpha-smooth muscle actin; MMPs = matrix metalloproteinases; FGF = fibroblast growth factor; ESR = erythrocyte sedimentation rate; PDGF = platelet-derived growth factor; CCLs = chemokine (C-C motif) ligands; MCPs = monocyte chemoattractant proteins; EGF = endothelial growth factor; IFN = interferon; iNOS = inducible nitric oxide synthase; eNOS = endothelial nitric oxide synthase; HIF-1α = hypoxia-inducible factor 1 alpha; Ki-67 = Kiel 67; NF-κB = nuclear factor kappa B; MPO = myeloperoxidase; TIMPs = tissue inhibitors of metalloproteinases; ROS = reactive oxygen species; p-ERK = phosphorylated Extracellular Signal-Regulated Kinase; IGF = Insulin-like growth factor).

Repeated Chemical Biomarkers	Sources			Study Categories		
	Freq.	% Included Sources	References	Freq.	% of Categories	Categories Included
ILs	50	25%	(42, 45, 48, 54, 57, 75, 80, 87, 91, 101, 103, 105, 107, 108, 110, 111, 117, 121, 124, 130, 135, 137, 138, 153, 156, 158, 161, 167, 170, 173, 176, 177, 180, 183, 187, 191, 194, 196, 198, 205, 206, 209, 212, 213, 215, 216, 223, 226, 227, 236)	7	54%	1, 3, 4, 9, 10, 11, 12
HbA1c	48	24%	(46, 58, 59, 61, 63, 67, 69, 72–74, 81, 83, 84, 90, 92, 94, 102, 109, 123, 133, 134, 140, 143–147, 152, 157, 159, 160, 162, 169, 170, 172, 181, 189, 195, 197, 198, 200, 203, 207, 222, 224, 228, 230, 234)	7	54%	1, 2, 3, 4, 6, 7, 8
VEGF	39	19%	(42, 48, 51, 52, 54, 56, 59, 70, 75, 76, 99, 103, 108, 112–115, 119, 121, 124, 125, 143, 148, 153, 158, 161, 165, 167, 177, 184, 186, 190, 191, 193, 204, 205, 209, 223, 236)	7	54%	1, 3, 5, 9, 10, 11, 12
CRP	34	17%	(46, 47, 65, 69, 74, 78, 82–84, 90, 92, 94, 102, 106, 123, 129, 133, 134, 145, 150, 152, 157, 160, 164, 168, 170, 172, 185, 198, 199, 209, 220,)	6	46%	1, 2, 6, 7, 8
TNF	34	17%	(42, 45, 54, 59, 71, 75, 76, 101, 103, 117, 121, 124, 135, 137, 153, 156, 158, 161, 167, 170, 173, 180, 191, 196, 198, 209, 212, 215, 216, 222, 223, 227, 232, 236)	6	46%	1, 3, 9, 10, 11, 12
Albumin	31	15%	(43, 44, 46, 58, 60, 63, 65, 77–79, 83, 84, 90, 102, 104, 106, 116, 133, 134, 157, 160, 161, 163, 172, 173, 179, 189, 198, 220, 234, 235)	5	38%	3, 6, 7, 8, 12
TGF	29	14%	(42, 51, 59, 70, 75, 76, 103, 113, 117, 121, 124, 132, 135, 137, 148, 158, 161, 165, 167, 170, 182, 183, 190, 191, 213, 215, 233, 236, 238)	6	46%	1, 3, 9, 10, 11, 12
WBC	27	13%	(44, 47, 59, 63, 77, 78, 82–84, 90, 94, 116, 123, 133, 134, 142, 152, 157, 159, 169, 172, 195, 220, 224, 230, 234, 235)	6	46%	2, 3, 4, 6, 7, 8
CD31	27	13%	(51, 55, 66, 71, 80, 93, 96, 103, 105, 107, 114, 119, 148, 165, 174, 177, 187, 190, 191, 194, 206, 208, 209, 211, 218, 219, 233)	3	23%	9, 10, 12
Hb	26	13%	(46, 47, 58, 61, 63, 69, 72, 78, 83, 84, 90, 94, 106, 116, 120, 133, 134, 145, 160, 168, 172, 189, 195, 197, 198, 217)	5	38%	2, 3, 6, 7, 8
α-SMA	23	11%	(54, 55, 71, 87, 88, 103, 113, 117, 119, 136, 148, 153, 174, 177, 178, 180, 190–192, 213, 215, 216, 233)	3	23%	9, 10, 12
MMPs	22	11%	(41, 45, 54, 59, 101, 113, 115, 167, 177, 191, 193, 202, 203, 211, 215, 216, 223, 226, 227, 232, 236, 239)	5	38%	3, 9, 10, 11, 12
Collagen	21	10%	(41, 54, 55, 59, 71, 75, 88, 105, 108, 113, 117, 137, 153, 191, 212–214, 216, 233, 236, 241)	6	46%	1, 3, 9, 10, 11, 12
Creatinine	20	10%	(77, 78, 84, 94, 116, 133, 134, 144, 157, 159, 161–163, 170, 173, 198, 199, 228, 234, 235)	6	46%	1, 3, 4, 6, 8, 12
Cholesterol	16	8%	(67, 69, 72, 74, 81, 84, 90, 92, 109, 133, 134, 170, 172, 224, 230, 234)	3	23%	1, 3, 6
FGF	16	8%	(41, 42, 49, 51, 98, 105, 107, 125, 136, 139, 148, 156, 166, 184, 191, 215)	5	38%	9, 10, 11, 12, 13
ESR	14	7%	(47, 59, 84, 94, 123, 133, 134, 157, 164, 198, 199, 207, 230, 235)	3	23%	1, 3, 6
CCLs/MCPs	12	6%	(42, 45, 108, 135, 148, 161, 177, 196, 206, 209, 212, 215)	3	23%	10, 11, 12
PDGF	11	5%	(42, 56, 76, 124, 125, 137, 141, 143, 161, 186, 236)	5	38%	3, 9, 10, 11, 12
Fasting Blood Sugar	11	5%	(61, 63, 67, 74, 84, 100, 133, 134, 143, 171, 230)	2	15%	3, 6
Neutrophils and Lymphocytes	11	5%	(47, 61, 63, 90, 129, 157, 171, 172, 179, 200, 224)	3	23%	3, 6, 7
EGF	10	5%	(41, 42, 56, 139, 143, 151, 166, 193, 198, 238)	4	31%	3, 10, 11, 13
Total Proteins	10	5%	(54, 92, 94, 117, 133, 134, 137, 185, 198, 234)	3	23%	3, 6, 9
Platelets	9	4%	(44, 47, 69, 79, 84, 133, 134, 142, 200)	3	23%	3, 6, 7
IFN	9	4%	(41, 45, 48, 91, 161, 173, 183, 198, 227)	4	31%	3, 10, 11, 12
iNOS and eNOS	9	4%	(71, 124, 167, 180, 191, 194, 206, 209, 226)	2	15%	9, 10
HIF-1α	7	3%	(48, 114, 119, 186, 191, 194, 236)	3	23%	3, 9, 10
Bacteria	7	3%	(61, 91, 115, 138, 201, 225, 227)	2	15%	3, 10
Ki-67	7	3%	(29, 51, 165, 177, 191, 219, 231)	2	15%	9, 10
NF-κB	6	3%	(48, 54, 117, 121, 167, 236)	2	15%	3, 9
MPO	6	3%	(121, 213, 216, 219, 227, 233)	2	15%	9, 10
Zinc	5	2%	(58, 104, 106, 133, 134)	2	15%	6, 8
TIMPs	5	2%	(45, 108, 115, 203, 236)	2	15%	3, 11
CD68	5	2%	(51, 117, 148, 155, 233)	2	15%	9, 12
ROS	5	2%	(49, 118, 165, 202, 218)	2	15%	9, 10
Hematocrit	4	2%	(44, 142, 152, 195)	2	15%	2, 7
ERK, p-ERK, and p-ERK1/2	4	2%	(29, 54, 113, 192)	2	15%	9, 10
IGF	4	2%	(48, 124, 177, 236)	2	15%	3, 10

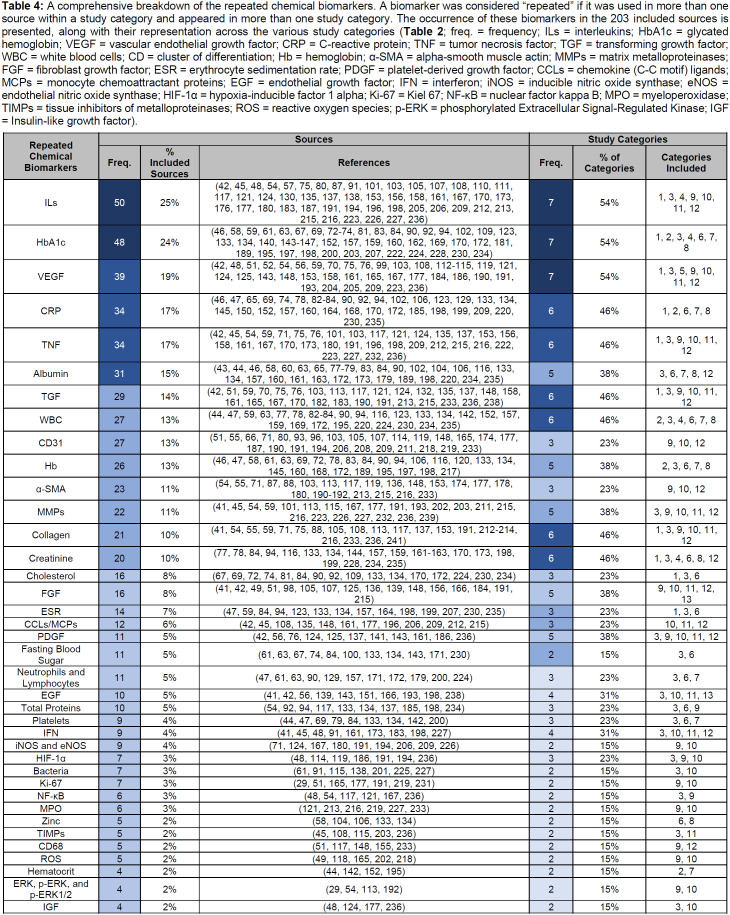

The distribution of repeated biomarkers across study categories (**Table 2** and **Table 4**) underscores the relationship between study design and biomarker prevalence. For instance, 27 of the 38 biomarkers were identified in bench research studies (Study Categories 9 to 13) which primarily use murine models (79 of 97 bench research included sources), indicating that such study types provide more detailed chemical biomarker data. In contrast, biomarkers exclusively observed in human participant studies only (Study Categories 1 to 8) include HbA1c, CRP, white blood cells (WBC), hemoglobin (Hb), cholesterol, erythrocyte sedimentation rate (ESR), fasting blood sugar, neutrophils and lymphocytes, platelets, zinc, and hemoglobin, all of which are typically routine blood biomarkers used to assess participants’ general health status. Additionally, the high prevalence of diabetic wound studies (explored in Study Categories 1 to 3, 6, and 9 to 11) is reflected in the extensive use of HbA1c, which is clinically utilized for diabetes diagnosis.^243^

### 3: MEASUREMENT TECHNIQUES OF THE REPEATED CHEMICAL BIOMARKERS

Gene expression was analyzed for 15 of the repeated chemical biomarkers using qRT-PCR (quantitative real-time polymerase chain reaction), including TaqMan assays (**Table 5** and **Table 6**). Immunostaining, to quantify biomarker expression in wound tissue samples, was similarly used for 15 of the 37 repeated biomarkers, such that qRT-PCR and immunostaining were the most frequently used quantification techniques. Interestingly, quantifying wound tissue biomarker expression used the greatest array of measurement techniques, including ELISA (enzyme-linked immunosorbent assay) kits, Western Blot, immunostaining, gelatine zymography, and multiplex immunoassays.

**Table 5:** Measurement techniques reported in included sources used to quantify gene expression, serum expression, and/or wound tissue expression of the identified repeated chemical biomarkers (ELISA = enzyme-linked immunosorbent assay; qRT-PCR = quantitative real-time polymerase chain reaction; biomarker abbreviations are defined in the **Table 4** caption).

	Biomarker Measurement Techniques										
	Gene Expression		Serum Expression		Wound Tissue Expression						
Repeated Chemical Biomarkers	qRT-PCR	TaqMan Assays	Routine Blood Test	ELISA Kit	Multiplex Immunoassay	ELISA Kit	Western Blot	Immunostaining	Gelatin Zymography	Multiplex Immunoassay	Luminol-Based Bioluminescence Imaging
Albumin			✓								
α-SMA								✓			
CCLs/MCPs	✓	✓		✓		✓		✓			
CD31								✓			
CD68								✓			
Cholesterol			✓								
Collagen	✓										
Creatinine			✓									
CRP			✓								
EGF						✓	✓	✓			
ERK, p-ERK, and p-ERK1/2	✓						✓	✓			
ESR			✓								
Fasting blood sugar			✓								
FGF	✓					✓		✓			
Hematocrit			✓								
Hb			✓								
HbA1c			✓								
HIF-1α	✓			✓			✓	✓			
IFN	✓			✓	✓					✓	
IGF	✓										
ILs	✓			✓		✓	✓				
iNOS and eNOS							✓	✓			
Ki-67								✓			
MMPs	✓	✓		✓		✓	✓	✓	✓		
MPO						✓		✓			
Neutrophils and Lymphocytes			✓								
NF-κB	✓					✓	✓				
PDGF	✓					✓		✓			
Platelets			✓								
ROS								✓			✓
TGF	✓			✓		✓					
TIMPs	✓	✓				✓	✓				
TNF	✓			✓		✓					
Total proteins			✓								
VEGF	✓			✓		✓	✓	✓			
WBC			✓								
Zinc			✓								
**Totals**	15	3	14	8	1	12	9	16	1	1	1
**% of 37 Biomarkers**	41%	8%	3%	22%	3%	32%	24%	43%	3%	3%	3%

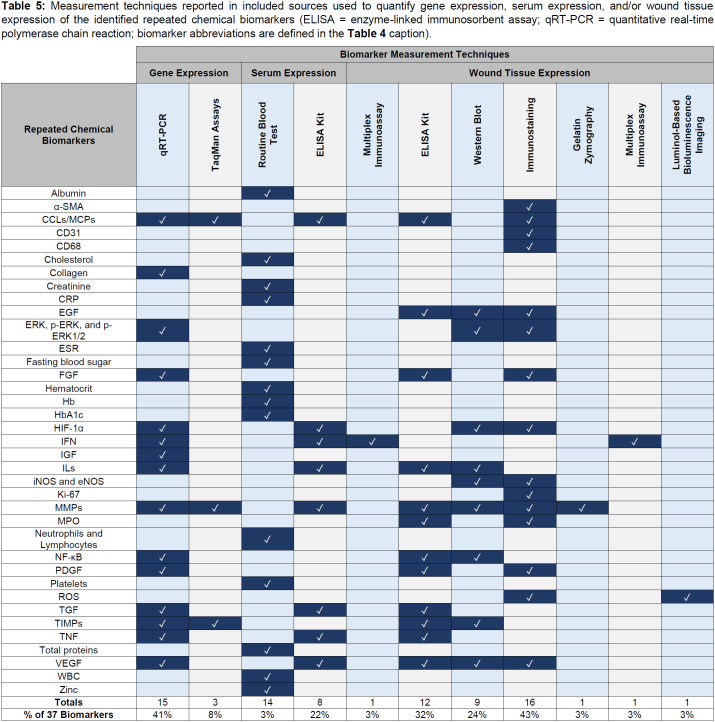

**Table 6:** Overview of the measurement techniques utilized in the included sources to quantify the chemical biomarkers referenced (**Table 4** (biomarkers); **Table 5** (corresponding quantification techniques); ELISA = enzyme-linked immunosorbent assay; MMP = matrix metalloproteinase; qRT-PCR = quantitative Real-Time Polymerase Chain Reaction; TaqDNA = Taq Deoxyribonucleic Acid Polymerase; miRNA = micro ribonucleic acid; BLI = bioluminescence imaging; NADPH = nicotinamide adenine dinucleotide phosphate; ROS = reactive oxygens species).

Chemical Biomarker Measurement Technique	Brief Description of Principle
Gelatin Zymography	Method to detect proteolytic enzymes capable of degrading gelatin from biological sources such as the gelatinases MMP-2 and MMP-9 (244).
ELISA	Employs the catalytic properties of enzymes to detect and quantify immunologic reactions (245). It is a solid-phase test generating a color reaction and is therefore easy to interpret (246).
Multiplex Immunoassay	It utilizes traditional immunoassay methods working on the principle of exploiting binder molecules (antibodies, proteins, or peptides) to capture circulating proteins or antibodies (246). Unlike ELISA, multiplex immunoassays enable the simultaneous measurement of multiple analytes in a single biological sample (246).
TaqMan Assay	This is a specific form of qRT-PCR and one of the earliest methods introduced for real-time PCR monitoring (247). It exploits the 5′ endonuclease activity of TaqDNA polymerase (an enzyme) to cleave an oligonucleotide probe during PCR, thereby generating a detectable signal (247).
Western Blot	Method to detect protein molecules among a mixture (248). The key steps include cell lysis (makes protein unfold into linear chains coated with a negative charge), gel electrophoresis (sorts proteins by size), blocking (prevents nonspecific reactions from occurring), incubating the sample with a primary antibody (binds specifically to the protein of interest), and finally incubating with a secondary antibody which binds to the primary and produces some signal (such as color or light) (248).
qRT-PCR	This is considered the gold standard for quantifying miRNAs with high sensitivity and specificity (249). It utilizes fluorescence generated during PCR to reflect the amount of DNA amplicons in a sample at a specific time (250).
Immunostaining	Requires incubating a tissue sample with antibodies specific to the protein of interest, which can then be visualized with a Requires incubating a tissue sample with antibodies specific to the protein of interest, which can then be visualized with a fluorescence (immunofluorescence) or chromogen (immunohistochemistry) which is bound to or binds to the antibody (251).
Luminol-Based Bioluminescence Imaging (BLI)	As demonstrated by Nguyen et al. (202), superoxide derived from NADPH oxidase can be detected through bioluminescence imaging by intraperitoneally injecting an animal with L-012. L-012 is a luminol-based chemiluminescent probe that emits light upon reacting with ROS (252). The intensity of the luminescent signal, measured in photons per second per centimeter squared, correlates with the amount of superoxide present, where a higher signal indicates greater superoxide levels, the most abundant ROS (253).

The number of measurement techniques for each biomarker varied. MMPs, for example, were assessed using 7 techniques, whereas markers found in the blood such as HbA1c (glycated hemoglobin), Hb (hemoglobin), WBC (white blood cells), and platelets were analyzed using only one method, a routine blood test.

Bacteria were assessed somewhat differently, as presented in the following list, and are therefore excluded from **Table 5**:

- Biofilms were detected using the tissue culture plate method.
- Antimicrobial susceptibility testing was performed using the Kirby-Bauer disc diffusion method.
- Molecular characterization of biofilm-forming resistant isolates was done by PCR.

## DISCUSSION

### 1: KEY FINDINGS

This scoping review identifies chemical biomarkers associated with the healing of tissues and structures in the residual limbs of adults with amputation. These biomarkers serve predictive, indicative, and diagnostic purposes, offering a foundation for improved prosthesis readiness and residuum health assessments.

Predictive biomarkers such as bacterial counts, nutritional markers (e.g., zinc, albumin), and routine blood markers (e.g., glycated hemoglobin [HbA1c], white blood cells [WBC], C-reactive protein [CRP]) indicate health status and help anticipate healing outcomes. For instance, elevated HbA1c is predictive of impaired healing due to hyperglycemia. Indicative biomarkers like growth factors, ILs, and reactive oxygen species (ROS) reflect critical healing processes, enabling monitoring of healing progression. Diagnostic biomarkers, such as alpha-smooth muscle actin (α-SMA), offer clear insights into wound healing at the cellular level.

Despite identifying 38 biomarkers in research, only routine blood markers are used clinically. Limited application stems from reliance on experimental methods (e.g., immunohistochemical staining) often restricted to animal studies. Bridging this gap requires advancements in measurement techniques that negate the need for wound tissue samples.

Population-specific factors (e.g., age, gender, comorbidities) and measurement differences (e.g., timing, location) influence healing and biomarker behavior. Thus, to improve healing assessment objectivity, a combination of biomarkers is required.

### 2: REPEATED CHEMICAL BIOMARKERS

#### 2.1: Chemical Biomarkers

To classify a biomarker as predictive, indicative, or diagnostic (**Table 7**), its role in the healing process and observed behavior in the reviewed sources must be considered. For example, interleukins (ILs), a class of cytokines predominantly expressed by leukocytes, are integral to inflammatory and immune responses^254^ and critical for wound healing. For instance, IL-2 receptors are present on macrophages, lymphocytes, keratinocytes, fibroblasts, vascular endothelial cells, and T-cells; cells that influence the entire healing process.^255^ Additionally, research on the treatment of diabetic foot ulcers (DFUs) with Therapeutic Magnetic Resonance (TMR^®^) devices revealed increased IL-10 expression and improved healing.^75^ Similarly, elevated IL-1RL2 and IL-33 gene expression is linked to inflammation and bone remodeling, suggesting predictive potential for healing post-percutaneous osseointegrated prosthesis implantation.^130^ Thus, ILs can be predictive, indicative, and diagnostic of healing.

**Table 7:** Classification of the repeated chemical biomarkers used in included sources as predictive, indicative, or diagnostic when considering their influence on the healing process and their behavior in the reviewed sources.

Biomarker		Predictive	Indicative	Diagnostic
Routine Blood Profile Biomarkers	Cholesterol (includes high and low-density lipoproteins and triglycerides)	✓		
	Erythrocyte Sedimentation Rate (ESR)	✓	✓	
	Fasting Blood Sugar (or Fasting Plasma Glucose)	✓		
	Glycated Hemoglobin (HbA1c)	✓		
	Hematocrit (HCT)	✓	✓	
	Hemoglobin (Hb)	✓		
	Neutrophils and Lymphocytes	✓		
	Platelets	✓		
	Total Proteins	✓	✓	
	White Blood Cell (WBC) Counts	✓		
Growth Factors	Epidermal Growth Factor (EGF)		✓	
	Fibroblast Growth Factor (FGF)		✓	
	Insulin-Like Growth Factor (IGF)		✓	
	Platelet-Derived Growth Factor (PDGF)		✓	
	Transforming Growth Factor (TGF)		✓	
	Tumor Necrosis Factor (TNF)		✓	
	Vascular Endothelial Growth Factor (VEGF)		✓	
Albumin		✓		
Alpha-Smooth Muscle Actin (α-SMA)			✓	✓
Bacteria (includes Colony-forming units, bacterial counts, and bacterial RNA assessment)		✓		
CC Chemokines (also known as Monocyte Chemoattractant [MCPs])			✓	
Clusters of Differentiation (CD68 and CD31)				✓
Collagen			✓	✓
C-Reactive Protein (CRP)		✓	✓	
Creatinine		✓		
Endothelial and Inducible Nitric Oxide Synthase (eNOS and iNOS)			✓	
Extracellular Signal-Regulated Kinases (ERKs)			✓	✓
Hypoxia Inducible Factor-1 (HIF-1)			✓	
Interferon (IFN)		✓	✓	
Interleukins (ILs)		✓	✓	✓
Kiel-67 (Ki-67)			✓	
Matrix Metalloproteinases (MMPs) and Tissue Inhibitors of Metalloproteinases (TIMPs)			✓	✓
Myeloperoxidase (MPO)			✓	
Nuclear Factor Kappa-Light-Chain-Enhancer of Activated B Cells (NF-κB)			✓	
Reactive Oxygen Species (ROS)			✓	
Zinc		✓		

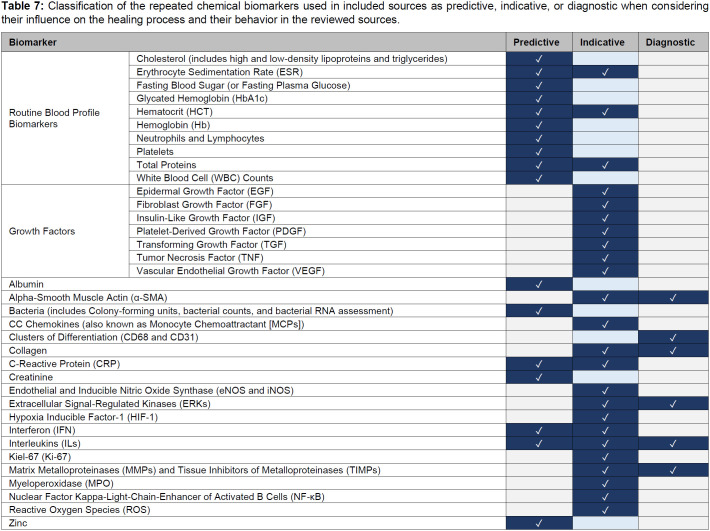

Predictive biomarkers, such as bacterial counts, nutritional markers (like zinc and albumin), and routine blood profile markers like glycated hemoglobin (HbA1c), white blood cell counts (WBCs), and C-reactive protein (CRP), indicate an individual's health status, enabling the anticipation of healing outcomes. For example, bacterial counts reflect the wound microbiome and potential infection, which impairs healing.^256^ Similarly, HbA1c indicates glycemic control^243^ and predicts healing, as hyperglycemia inhibits keratinocyte migration and promotes oxidative stress through reactive oxygen species (ROS) production.^257^ Zinc is a marker of nutritional status,^258^ with deficiency negatively impacting healing,^259^ and supplementation accelerating it.^260^ Predictive biomarkers primarily identify comorbidities or conditions, such as infection or poor nutritional status, that contribute to impaired healing rather than diagnosing specific healing mechanisms. This makes them appropriate for pre-amputation risk assessments, given the high prevalence of comorbidities, such as diabetes, among individuals undergoing amputation. For example, the Scottish Physiotherapy Amputee Research Group (SPARG) reported in 2019 that 56% of lower limb amputees recorded had the etiology of diabetes.^261^

Indicative biomarkers, including growth factors (**Table 7**), ILs, and signaling molecules like ROS and nuclear factor kappa-light-chain-enhancer of activated B cells (NF-κB), reflect biological processes essential for healing (e.g., tissue remodeling and cellular proliferation), enabling monitoring and quantification of progress. For example, vascular endothelial growth factor (VEGF) promotes angiogenesis by influencing vascular endothelial cells, keratinocytes, and macrophages.^262^ Supporting this, Kim et al.^103^ demonstrated that increased VEGF levels correlated with near-complete epithelial coverage in a diabetic wound mouse model treated with Substance P, indicating healing.

The 6 identified diagnostic biomarkers (**Table 7**) provide insights into tissue structure and composition, enabling precise healing assessments. For example, alpha-smooth muscle actin (α-SMA), expressed temporarily by myofibroblasts during their differentiation from granulation tissue fibroblasts,^263^ serves as a marker of smooth muscle differentiation and wound contraction,^264^ diagnosing healing progression during the epithelialization phase. By directly reflecting healing at a cellular level, diagnostic biomarkers offer the most objective insights into healing progression and hold high objective value for inclusion in a post-amputation healing assessment scale.

While numerous biomarkers show promise for enhancing post-amputation healing, their clinical application requires careful categorization and validation. Indicative biomarkers, such as growth factors, also diagnose specific molecular mechanisms, suggesting potential as diagnostic tools. For example, Ki-67, a marker of cellular proliferation,^26,27^ plays a diagnostic role by identifying fibroblast, endothelial cell, and keratinocyte proliferation (key processes in cutaneous wound healing).^28^ However, further research in amputeespecific populations is needed to validate such biomarkers for clinical use, facilitating their transition from bench research to diagnostic applications.

Many identified biomarkers, including ILs and WBCs, are integral to immune and inflammatory responses. Elevated WBC counts (leukocytosis), for instance, are linked to higher risks of re-amputation, longer post-amputation healing times, and greater chance of amputation due to DFUs.^44,84,235^ However, leukocytosis may stem from factors unrelated to wound healing, such as infections elsewhere, medications, stress, or serious conditions like leukemia.^265^ To address this variability in biomarker causation, a broader array of biomarkers is needed to capture all phases of healing and account for patient-specific factors known to affect healing like stress,^266^ poor nutrition,^259^ renal disease,^267^ smoking,^268,269^ and alcohol use.^270^

The limitations of predictive biomarkers are evident in conflicting findings. To illustrate, Adams et al.^43^ reported higher mortality rates after transmetatarsal amputation (TMA) in patients with preoperative albumin levels below 3.5 g/dL (p < 0.05). Similarly, Brookes et al.^58^ observed significantly lower albumin levels in amputees compared to non-amputees (p = 0.03). However, Ahn et al.^44^ found no significant correlation between serum albumin and TMA re-amputation rates (p = 0.644). Although the sources differ in participant populations and follow-up durations, the contrasting conclusions highlight the need for a biomarker profile rather than relying on a single predictive biomarker. This will enhance predictive accuracy whilst acknowledging a biomarker's limitations.

Future research must clarify the impact of quantification timing and location on biomarker levels during healing, to optimize their clinical application. For example, Anguiano-Hernandez et al.^48^ demonstrated that NF-κB expression and localization are indicative of healing progression. In DFU patients treated with hyperbaric oxygen therapy, NF-κB expression decreased, and its localization shifted from nuclear to cytoplasmic in endothelial cells and fibroblasts, correlating with complete healing.^48^ These findings stress the importance of not only measuring biomarker levels but also assessing their localization to fully understand their role in the healing process.

A comprehensive biomarker profile that spans predictive, indicative, and diagnostic categories is essential to enhance the assessment and management of post-amputation healing. Such profiles would account for comorbidities, capture all healing stages, and improve clinical decisionmaking, particularly in early prosthetic rehabilitation.

#### 2.2: Quantification Techniques

The method by which a biomarker is quantified dictates its applicability in research and clinical settings. Diagnostic markers like Ki-67, CDs (clusters of differentiation), α-SMA, and ERKs (extracellular signal-regulated kinases) rely on techniques such as immunohistochemical staining, immunofluorescence, or RT-qPCR (**Table 5**), which require tissue samples, making them time-consuming, costly, and ethically challenging in human studies. Consequently, their use is largely confined to murine models emphasizing the need for advancements in quantification techniques. For example, ROS can be quantified via non-invasive in vivo chemiluminescence imaging,^202^ validated in animal models^271^ but untested clinically.

Conversely, biomarkers like CRP and routine blood markers, measurable through peripheral blood draws,^272^ are more feasible for human studies and already employed in clinical settings.^273^ Unfortunately, such biomarkers are typically predictive or indicative, whereas markers like Ki-67 and α-SMA are diagnostic and thus hold greater clinical value.

Developing accessible, cost-effective, and ethically viable quantification techniques will facilitate the integration of chemical biomarkers into research and clinical practice, ultimately optimizing post-amputation healing and prosthetic rehabilitation outcomes.

### 3: OVERALL SEARCH RESULTS AND STUDY CHARACTERISTICS

Trends in study characteristics align with the findings from the Part 2 review;^18^ for full details, refer to Part 2. Diabetic wounds dominated the reviewed sources, reflecting the global diabetes burden,^274^ with DFUs being a major diabetic complication^275^ and a risk factor for amputation,^276,277^ reinforcing the importance of pre-amputation biomarker assessments to identify comorbidities predictive of non-healing like diabetes.^8^

Aging further complicates the healing process, with the median mean age of participants in Study Categories 1 to 8 ranging from 58.1 to 72.0 years. Non-healing wounds are often linked to vascular disease,^278^ venous insufficiency,^279^ areas of high unrelieved pressure,^280^ diabetes,^281^ and disability;^282^ conditions that are increasingly prevalent as the population ages.^281^ Aging contributes to prolonged inflammation and increased ROS production,^283^ necessitating objective measures to monitor wound healing, particularly for older adults requiring prosthetic fitting.^261^

Gender differences were evident, with male participants at higher risk for DFU development,^284^ poorer DFU healing,^285^ increased post-surgery infection rates,^286^ and higher inhospital immortality rates after trauma.^287^ This highlights the need for gender-specific research^288^ and biomarkers unaffected by hormonal or gender-related factors.

Most included sources investigated wound healing in populations similar to individuals with amputation rather than residual limb healing specifically, highlighting the lack of standardized approaches and the need for a foundational database of biomarkers for residual limb recovery, particularly for lower limbs, which have unique health requirements due to weight-bearing during ambulation.

### 4: METHODOLOGICAL DISCUSSION

#### 4.1: Methodological Strengths

This review's methodology aligns with Part 1 and Part 2; detailed discussions of methodological strengths, limitations, and ethical considerations can be found there.

This review broadly explored chemical biomarkers associated with post-amputation healing, serving as a foundation for future systematic reviews on specific biomarkers supported by high-quality evidence. A key strength is its focus on diagnostic, predictive, and indicative biomarkers with the potential to improve the prevention and treatment of non-healing surgical sites and to enhance post-amputation healing assessment, enabling timely prosthetic interventions.^289^

#### 4.2: Methodological Limitations

Limitations include the unreliability of animal studies due to biological differences^290^ and the oversimplification of human biology in mathematical models,^291,293^ requiring cautious interpretation of biomarker behavior reported in these source types. While the review included wound types relevant to the residuum, future research should differentiate between the healing of secondary intention wounds (e.g., DFUs) and primary intention wounds (e.g., surgical sites). Additionally, prioritizing only repeatedly studied biomarkers risks oversimplification.

### 5: ETHICAL CONSIDERATIONS

Ethical rigor was prioritized over strict adherence to evidence hierarchies, such that only studies with clear ethical approval and informed consent from participants aged 18 or older were included. Grey literature was reviewed to reduce bias,^294^ but none met the inclusion criteria due to methodological shortcomings and lack of ethical transparency.

## CONCLUSION

This scoping review identified 38 repeated chemical biomarkers relevant to healing in the tissues and structures in residual limbs of adults with amputation, classified as predictive, indicative, or diagnostic based on their function and behavior in the 203 reviewed sources. Predictive biomarkers, such as blood markers (e.g., glycated hemoglobin [HbA1c], white blood cells [WBC]), assess health and healing potential, aiding pre-amputation risk assessments and identifying conditions impairing healing, like infection or poor nutrition. Indicative biomarkers, including growth factors and interleukins (ILs), reflect biological processes like cell proliferation and tissue remodeling, essential for post-amputation healing. For instance, vascular endothelial growth factor (VEGF) supports angiogenesis (blood vessel formation), a key healing component. Diagnostic biomarkers, such as alpha-smooth muscle actin (α-SMA), reveal tissue structure and healing progress at the cellular level.

While many biomarkers show potential for improving post-amputation healing, their clinical application requires careful validation in amputee populations. Biomarkers like WBCs play a key role in immune responses, but elevated WBC counts can be influenced by factors unrelated to wound healing, such as infections or stress. Using a biomarker array could better capture all healing stages and account for comorbidities, population differences, and lifestyle factors (e.g. infection, poor nutrition, smoking, alcohol use, gender, and age) known to affect healing. Understanding the impact of biomarker quantification, timing and location (e.g. wound fluid or serum) is crucial for clinical optimization.

Integrating diagnostic biomarkers into clinical practice is challenged by the invasive and complex nature of current measurement techniques. Most biomarkers, apart from routine blood markers like cholesterol and WBC counts (which are predictive of healing), remain confined to research due to reliance on techniques like immunohistochemistry requiring tissue samples, raising ethical and logistical barriers. Further research must develop accessible, non-invasive diagnostic tools. Bridging the gap between experimental research and clinical application is essential to standardize post-amputation healing assessments, reduce subjectivity, and ultimately enhance patient rehabilitation outcomes.

## Abbreviations & Acronyms:

**Abbreviations & Acronyms: Definition**
BLI: Bioluminescence Imaging
CCL: Chemokine (C-C motif) Ligand
CD: Cluster of Differentiation
CRP: C-Reactive Protein
DFU: Diabetic Foot Ulcer
DNA: Deoxyribonucleic Acid
EGF: Epidermal Growth Factor
ELISA: Enzyme-Linked Immunosorbent Assay
ELISA: Enzyme-Linked Immunosorbent Assay
eNOS: Endothelial Nitric Oxide Synthase
ERK: Extracellular signal-Regulated Kinase
ESR: Erythrocyte Sedimentation Rate
FBS: Fasting Blood Sugar
Freq.: Frequency
FGF: Fibroblast Growth Factor
Hb: Hemoglobin
HbA1c: Hemoglobin A1C (glycated hemoglobin)
HCT: Hematocrit
HDL: High-Density Lipoprotein
HIF1-α: Hypoxia-Inducible Factor 1 Alpha
IFN: Interferon
IGF: Insulin-like Growth Factor
IL: Interleukin
iNOS: Inducible Nitric Oxide Synthase
JBI: Joanna Briggs Institute
Ki-67: Antigen Kiel 67
LDL: Low-Density Lipoprotein
MCP: Monocyte Chemoattractant Protein
MMP: Matrix Metalloproteinases
MPO: Myeloperoxidase
NA: Not Applicable
NADPH: Nicotinamide Adenine Dinucleotide Phosphate
NF-κB: Nuclear Factor kappa-light-chain-enhancer of activated B cells
No.: Number
PDGF: Platelet-Derived Growth Factor
PRISMA-ScR: Preferred Reporting Items for Systematic Reviews and Meta-Analyses extension for Scoping Reviews
qRT-PCR: Quantitative Reverse Transcription Polymerase Chain Reaction
RCT: Randomized Controlled Trial
Refs.: References
RNA: Ribonucleic Acid
ROS: Reactive Oxygen Species
SPARG: Scottish Physiotherapy Amputee Research Group
TGF: Transforming Growth Factor Tissue Inhibitor of Metalloproteinase
TIMPs: Tissue Inhibitor of Metalloproteinase
TMA: Transmetatarsal amputation
TMR^®^: Therapeutic Magnetic Resonance
TNF: Tumor Necrosis Factor
USA: United States of America
VEGF: Vascular Endothelial Growth Factor
WBC: White Blood Cells
α-SMA: Alpha-Smooth Muscle Actin
